# Reduction of False-Positive Markings on Mammograms: a Retrospective Comparison Study Using an Artificial Intelligence-Based CAD

**DOI:** 10.1007/s10278-018-0168-6

**Published:** 2019-04-08

**Authors:** Ray Cody Mayo, Daniel Kent, Lauren Chang Sen, Megha Kapoor, Jessica W. T. Leung, Alyssa T. Watanabe

**Affiliations:** 1 Department of Diagnostic Imaging, Breast Imaging Division, MD Anderson Center, University of Texas, 1515 Holcombe Blvd., Houston, TX 77030 USA; 2 Voxel Imaging, Inc., 2711 N. Sepulveda Blvd., #284, Manhattan Beach, CA 90266 USA; 3 Keck School of Medicine, University of Southern California, 1975 Zonal Avenue, Los Angeles, CA 90033 USA

**Keywords:** Breast imaging, Mammogram, Computer-aided detection, False-positive exam, Artificial intelligence

## Abstract

The aim was to determine whether an artificial intelligence (AI)-based, computer-aided detection (CAD) software can be used to reduce false positive per image (FPPI) on mammograms as compared to an FDA-approved conventional CAD. A retrospective study was performed on a set of 250 full-field digital mammograms between January 1, 2013, and March 31, 2013, and the number of marked regions of interest of two different systems was compared for sensitivity and specificity in cancer detection. The count of false-positive marks per image (FPPI) of the two systems was also evaluated as well as the number of cases that were completely mark-free. All results showed statistically significant reductions in false marks with the use of AI-CAD vs CAD (confidence interval = 95%) with no reduction in sensitivity. There is an overall 69% reduction in FPPI using the AI-based CAD as compared to CAD, consisting of 83% reduction in FPPI for calcifications and 56% reduction for masses. Almost half (48%) of cases showed no AI-CAD markings while only 17% show no conventional CAD marks. There was a significant reduction in FPPI with AI-CAD as compared to CAD for both masses and calcifications at all tissue densities. A 69% decrease in FPPI could result in a 17% decrease in radiologist reading time per case based on prior literature of CAD reading times. Additionally, decreasing false-positive recalls in screening mammography has many direct social and economic benefits.

## Introduction

Any method to improve the performance of mammography interpretation could tremendously affect patient care, radiologist workflow, and system costs. Multiple studies in the early and mid-2000s showed variable ability of computer-aided detection (CAD) to improve diagnostic performance [1–10]. However, more recent studies show that CAD may not improve the diagnostic ability of mammography in any performance metric including sensitivity, specificity, positive predictive value, recall rate, or benign biopsy rate [11, 12]. Several recent studies have even shown an increase in callback rates and an increase in false-positive recalls from screening after implementation of CAD [11].

The largest disadvantage of using currently available CAD systems is the high rate of false-positive marks. One metric to assess the usefulness of CAD is the count of these marks on each image—false positives per image (FPPI). False-positive marks may distract the interpreting radiologist with too much “noise” and could lead to unnecessary workups and biopsies [13]. Therefore, high FPPI is a common complaint of radiologists when reviewing CAD marks.

Approximately $4 billion dollars per year are spent in the USA on false-positive recalls and workups. The cost for diagnostic mammogram workups alone is 1.62 billion [14]. For the patient, these false-positive screening mammograms create unnecessary anxiety and may lead the patient to a biopsy that was not needed. Diagnostic mammograms also take longer to perform and interpret than screening studies but carry almost no increase in reimbursement. Any reduction in false-positive screening exams would directly increase accuracy and lower costs which would result in improved outcomes for the patient, payor, and physician [15]. A reduction of false-positive mammograms therefore represents a desirable and objective target for improvement.

Within the last 5 years, the field of artificial intelligence (AI) has undergone tremendous advancement with increasing influence throughout the scientific and engineering communities. Image analysis was initially slow to develop given its complexity; however, advanced analytics such as deep learning and convolutional neural networks are driving new waves of progress. The result is a new generation of AI algorithms capable of imaging analysis. One of these which is designed to assess mammograms is evaluated in this paper.

In the same way, a radiologist’s performance can vary depending on complexity of cases; CAD software will also show variations in performance across different datasets. Currently, CAD programs are tested on proprietary internal data sets for assessment. In order to have a meaningful comparison, a head-to-head trial where both are tested on the same dataset is needed. Our study compares the performance of a recently developed AI-CAD algorithm directly to a commercially available conventional CAD software using the same test dataset of clinical cases. To our knowledge, this is the first published study in the peer reviewed literature comparing the FPPI of an AI-CAD to a conventional CAD using the same test set.

## Materials and Methods

### Study Population

A retrospective study was performed on a set of 250 two-dimensional (2D) full-field digital mammograms (FFDM) collected from a tertiary academic institution based in the USA which specializes in cancer healthcare. All of the mammograms were originally interpreted using the ImageChecker CAD, version 10.0 (Hologic, Inc., Sunnyvale, CA).

Authors maintained control of the FFDM data and protected health information submitted for publication throughout the study. As no diagnostic or therapeutic intervention was involved, and no direct patient contact was required for this retrospective chart review, a waiver was granted by the hospital’s institutional review board (IRB). All of the mammograms were anonymized using a Health Insurance Portability and Accountability Act (HIPAA)-compliant protocol.

Inclusion criteria were female asymptomatic patients of all ages and of any race with 2D screening mammograms performed at the academic institution between January 1, 2013, and March 31, 2013, and whose mammogram records contain archived CAD markings. Mastectomy and breast implant patients were excluded from analysis.

### Case Selection

All patients in this study were females aged 40–90 years who had a screening mammogram performed between January 2013 and March 2013. Of the 250 cases reviewed, five were eliminated from the study due to mastectomies. The remaining data set of 245 mammograms included standard four-view studies which were comprised of 224 BI-RADS assessment category 1 or 2 patients, and 21 BI-RADS assessment category 0 patients. Ultimate truth status was determined by biopsy or 2+ years of follow-up. Three cases were proven to be malignant. This date selection provides enough time for follow-up cancer diagnosis data to determine true-positive, false-positive, true-negative, and false-negative rates.

### Products Compared

This retrospective study compared cmAssist (prototype AI-CAD, CureMetrix, La Jolla, CA) to ImageChecker (version 10.0 software, Hologic, Sunnyvale, CA), a currently available FDA-approved conventional CAD. The default setting for the CAD (operating point 1 for calcifications, and operating point 2 for mass) was used for this study. The AI-CAD software was also set at the default operating points for mass and calcifications (operating point 2 for both). The default operating points used for both softwares are considered to be their optimal settings by the manufacturers.

The number, location, and type of lesions (either mass or calcification) marked by the CAD were recorded and compared to the lesions marked by the AI-CAD. Asymmetries were classified as masses for this study. All images and medical record data including pathology data were reviewed by one of the three Mammography Quality Standards Act (MQSA) certified, breast imaging fellowship-trained radiologists. For the biopsied cases, one of the radiologists created a region of interest (ROI) circle to establish the location of the biopsied lesion. The CAD and AI-CAD marks of all cases in the test set were then compared by the radiologists to the truth data to classify whether the marks were false positive or true positive. The biopsy-validated lesions were categorized as correctly marked if the geometric center of the CAD mark lay within the ROI marking generated by the radiologist.

### Data Analysis

False positives per image (FPPI) was the primary metric used to compare the two different products in this study. Each set of mammograms was analyzed with both programs CAD and AI-CAD and the number of marks was counted. FPPI was calculated for multiple different categories: normal cases (BI-RADS 1 or 2), recalled cases (BI-RADS 0), mass marks, calcification marks, and tissue densities. Additionally, cases with no markings on any images within a case were also counted. Sensitivity was measured as the number of true positive cases where at least one lesion is correctly marked in at least one view. Specificity was measured as the number of normal and actionable benign cases without marks divided by the total number of normal and actionable benign cases. On this dataset, specificity for CAD was calculated at 16% (39/242) and 47% (113/242) for AI-CAD; or roughly three times better for AI-CAD than CAD.

Confidence intervals (CI) at 95% were calculated for the FPPI and case-based false-positive rate (FPR) of AI-CAD results to determine statistical significance. The 95% CI is obtained using the bootstrap statistical analysis [16] with 10,000 samples. In this technique, each sample consists of 242 cases selected *randomly* from the original 242 normal/biopsy-benign cases in the dataset. Note that due to the random selection, a given case may be absent while others may appear several times in a given sample. The FPPI and FPR are computed for each sample, and the mean and standard deviation (for FPPI and FPR) are obtained from the set of 10,000 samples. The 95% CI corresponds to [− 1.96, + 1.96].

## Results

There were 21 recalled cases (BI-RADS 0) from the 245 included cases which equates to a recall rate of 8.6%. The tissue density distribution was 3% fatty, 46% scattered, 48% heterogeneously dense, and 2% extremely dense. Each of the three cancer cases was marked correctly by both CAD and AI-CAD, which equates to 100% sensitivity for both.

**Fig. 1:**
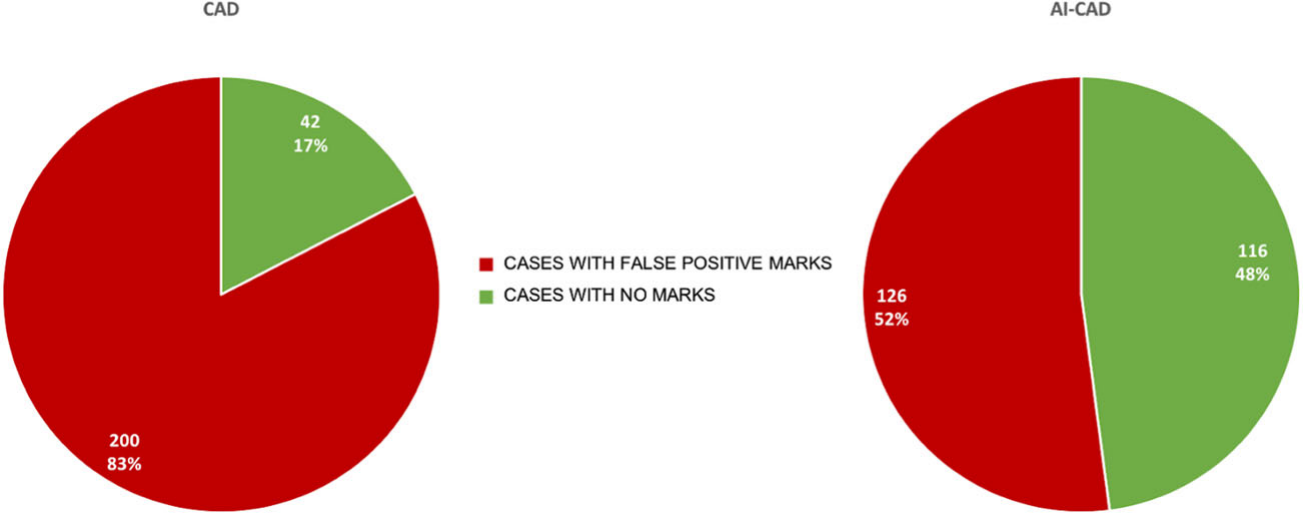
CAD (left) and AI-CAD (right). Graphs show the number and percentage of cases that show false-positive marks and number and percentage of cases with no false marks. Only 17% of CAD cases were mark-free compared to 48% of the AI-CAD cases

CAD displayed false marks on 200 of the 242 non-cancer cases (83%) and no marks at all on 42 cases (17%). AI-CAD software displayed false marks on 126 cases of the 242 non-cancer cases (52%) and no marks at all on 116 cases (48%) (Fig. 1). This equates to 37% fewer cases with false marks with AI-CAD and simultaneously 64% more mark-free cases with AI-CAD compared with CAD.

**Fig. 2:**
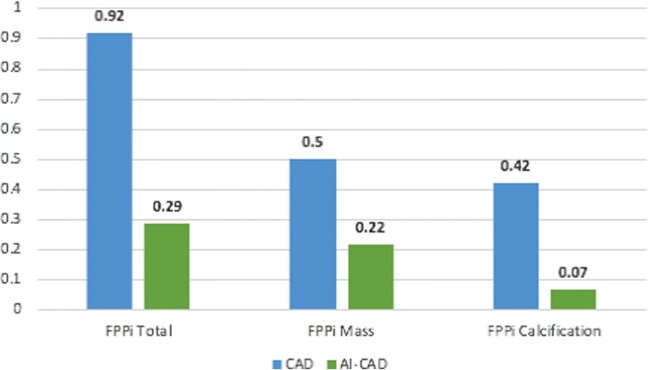
FPPI (false positive per image). There is a significant 69% reduction in FPPI using AI-CAD (green bar) compared to CAD (blue bar). The AI-CAD FPPI reduction was significant for both mass and calcification marks

There was a 69% reduction in overall FPPI with AI-CAD compared to CAD. Evaluation by lesion type shows outperformance of AI-CAD for both masses and calcifications. There was an overall 83% reduction in FPPI for calcifications with AI-CAD and 56% reduction for mass. FPPI for masses was higher than FPPI for calcifications for both systems (Fig. 2).

The overall FPPI for AI-CAD is 0.29, with 95% confidence interval (CI) of range 0.21–0.35. The overall FPPI for CAD is 0.92, well outside of the 95% CI. For calcifications, AI-CAD’s FPPI is 0.07 (with 95% CI range of 0.041–0.11) compared to CAD’s FPPI of 0.42. For masses, AI-CAD’s FPPI is 0.22 (with 95% CI range of 0.18–0.26) compared to CAD’s FPPI of 0.50. There was a 69% reduction in overall FPPI with AI-CAD compared to CAD. Evaluation by lesion type shows outperformance of AI-CAD for both masses and calcifications. There was an overall 83% reduction in FPPI for calcifications with AI-CAD and 56% reduction for mass. FPPI for masses was higher than FPPI for calcifications for both systems (Fig. 2).

The reduction in false-positive markings per image was consistent across all tissue densities from fatty to extremely dense with AI-CAD. The reduction in FPPI with AI-CAD was also demonstrated for both mass and calcification categories. For cases that were ultimately benign, CAD showed 39 of 242 without marks and AI-CAD showed 113. This results in a specificity of a case without flags of 16% for CAD and 47% for AI-CAD. With the use of CAD, twenty-one cases were categorized as BI-RADS 0 and recalled from screening (Fig. 4). Of these, 18 cases (86%) were false-positive recalls and were subsequently confirmed to be benign through biopsy or long-term follow-up of 2 years or more. Fifteen of the 18 false-positive cases had CAD marks whereas only 8 of the 18 had AI-CAD marks.

## Discussion

Ultimately, the radiologist will make the final decision regarding BIRADS assessment, but their decisions are influenced by CAD marks. In order to reduce false-positive BIRADS assessments, the most desired improvement for imaging analysis software in mammography today is reduction in FPPI. In our study, 83% of the false-positive BIRADS 0 cases had CAD marks, whereas only 56% of these cases had AI-CAD marks.

The significant reduction in FPPI with AI-CAD may translate into fewer false recalls, improved workflow, and decreased costs. The economic impact of the false-positive recall is considered to be one of the major drawbacks of screening mammography. Fewer recalled patients means more availability to address backlogs for both screening and diagnostic appointments. Departmental screening throughput may be increased without adding staff, equipment, or facility hours since typically two screening exams are often allocated the same time as one diagnostic exam on the schedule.

**Fig. 3:**
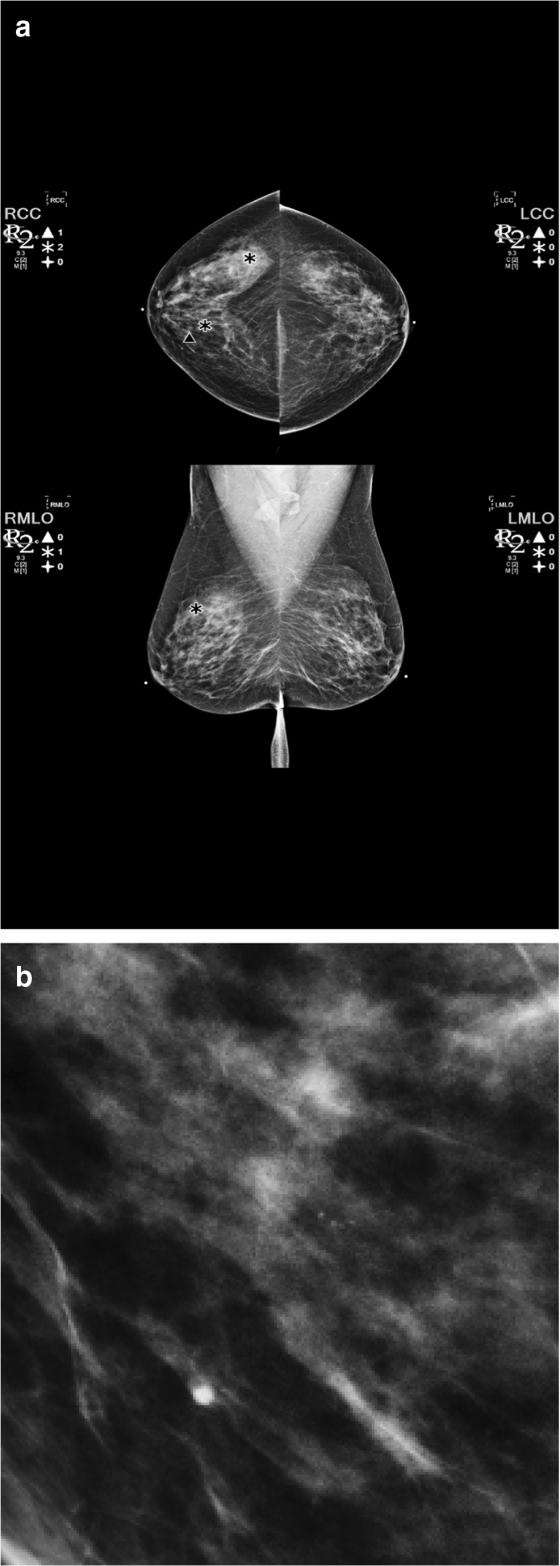
**a** Bilateral screening mammogram with CAD marks: There are multiple CAD marks in the right breast. The patient was recalled and underwent a stereotactic biopsy for calcifications (marked by CAD triangle) on right CC view. Results were benign. The AI-CAD did not mark any abnormalities in this case. **b** Enlargement of the right CC mammogram shows the benign calcifications that were marked by CAD but not AI-CAD. This biopsy could have been avoided if the recall was due to the CAD false mark.

There are known psychological and physical risks for patients related to false-positive recalls [17]. The scare of a potential of a breast lesion can be a frightening experience. Even when the workup results are benign, the psychological effects from anxiety can last up to 3 years [18]. Patient recalls also reduce workforce productivity due to missed work. Figure 3 illustrates a small group of calcifications that were marked by CAD. The patient underwent a stereotactic biopsy which yielded sclerosing adenosis. These benign calcifications were not marked by AI-CAD. Forty-seven percent of the benign biopsies in this study showed no false-positive AI-CAD flags. It is possible that some of these biopsies could have been avoided if the recall was due to the influence of the false CAD marks.

Radiologist efficiency is extremely important today in the era of productivity metrics, rising volumes, radiologist burnout, and shortage of qualified mammographers. It takes radiologists longer to read cases with CAD marks than without CAD marks. In one study with 12 readers, the median reading time per case without CAD was 44.4 s and with CAD was 55.6 s, or an increased average of 11.2 s in reading time [19]. This resulted in a 22% increase in average reading time for cases with CAD. Another study showed that reading a screening mammogram with CAD marks on average added an additional 23 s to the reading time. In that study, on average it took an extra 7.3 s to review each mass flag and an extra 3.2 s for each calcification flag [20]. This equated to a 6% increase in reading time for every marked mass and a 3% increase in reading time for every marked calcification. Using this data, the increased reading time per image with CAD for these 245 cases would be 4.97 s versus 1.81 s with the AI-CAD (based on FPPI = 0.92 for CAD and FPPI = 0.29 for AI-CAD). When considering a four-view screening mammogram, 19.9 additional seconds are required to a study with CAD compared with additional 7.2 s with AI-CAD. This represents a 64% decrease in reading time for AI-CAD compared to CAD.

The AI-CAD in this study is heavily influenced by deep learning technology, which is the most popular form of AI used today. The AI-CAD was trained with radiologist curated mammograms that contained both validated benign and malignant lesions. These cases known as “truth data” were evaluated by the software and the AI part of the algorithm “learned” to recognize different features of these lesions through the use of convolutional neural networks (CNN). CNN is a form of artificial neural network that is commonly applied to image analysis technology. The AI-CAD assigns a unique data-driven NeuScore (™) which is a quantitative measure of suspiciousness of a region of interest ranging from 0 (least suspicious) to 100 (highly suspicious). If the NeuScore exceeds a certain threshold value, it will appear on the mammogram as an AI-CAD mark. This threshold is determined by setting the software operating point, and this study used the default operating point for the AI-CAD program. The NeuScore is an additional benefit of AI-CAD that CAD cannot provide.

**Fig. 4:**
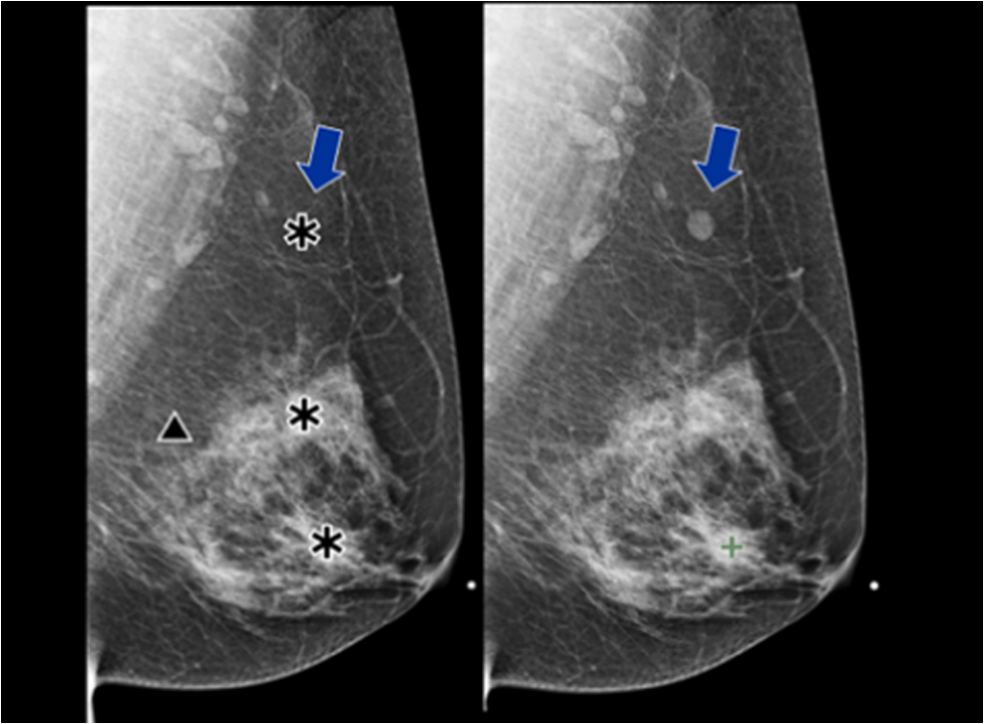
Left MLO mammogram with CAD (left) and AI-CAD (right). There are multiple false marks by CAD including a benign axillary lymph node (blue arrow) which was not marked by AI-CAD (blue arrow). Through AI self-learning feature, AI-CAD is trained not to mark most benign lymph nodes which results in a significant reduction in FPPI

**Fig. 5:**
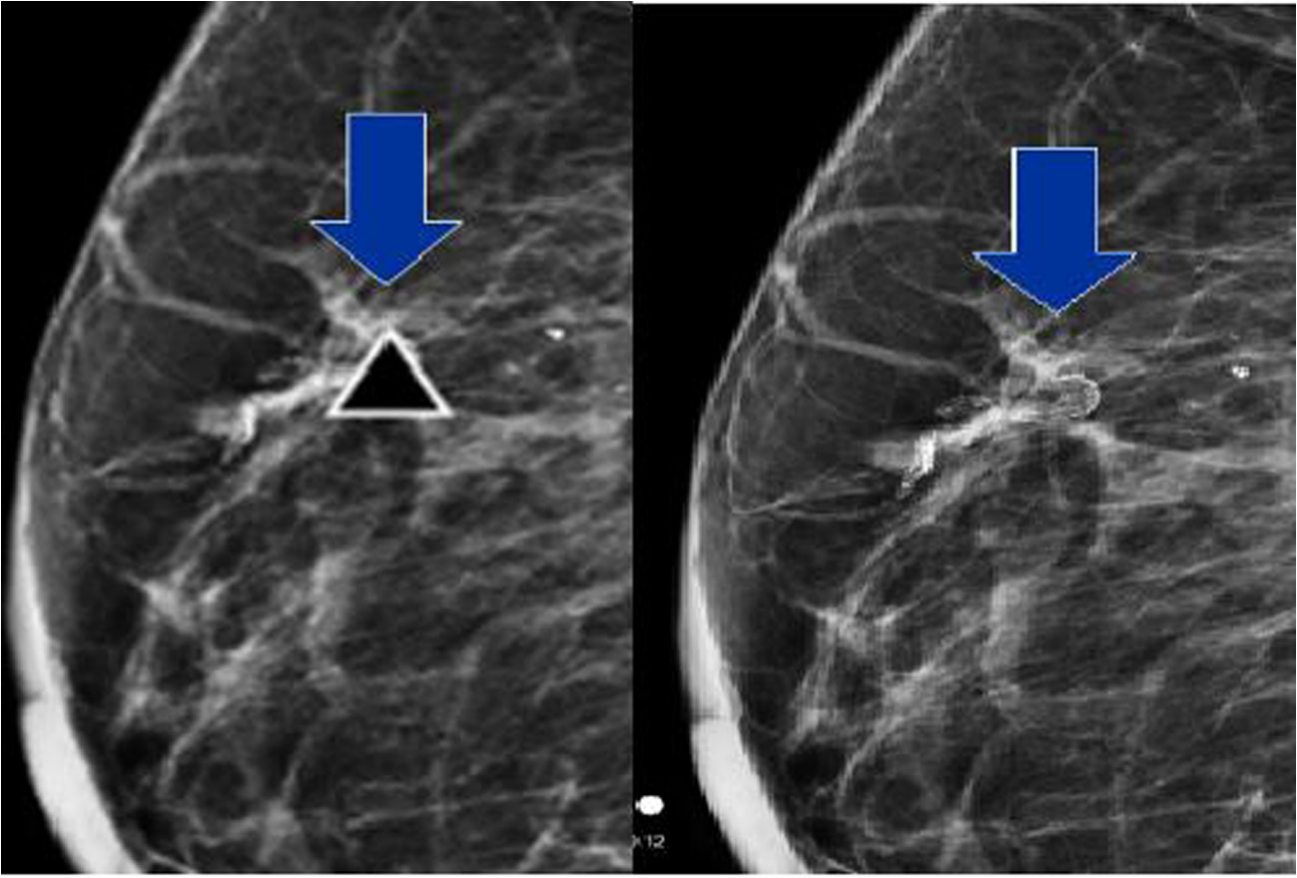
Enlarged RMLO mark-up images for CAD (left) and AI-CAD (right). False CAD mark (black triangle) of vascular calcifications (blue arrow) on the left and AI-CAD on the right without a flag. Elimination of false-positive marks reduces distractions to the radiologist and also reduces radiologist reading time

Figure 4 shows a case of a benign axillary lymph node which is marked by CAD but not marked by AI-CAD. Figure 5 shows an example of typical benign arterial calcifications which are marked by the CAD but not marked by AI-CAD. These commonly seen benign findings are frequently marked by CAD, but are rarely marked by AI-CAD through deep learning training. As more training data is entered, it is expected that AI-CAD, through its self-learning feature, will become increasingly accurate at categorizing benign versus malignant lesions. In addition, the use of the NeuScore ™ can potentially be employed by radiologists to further enhance their clinical decision-making. It has been shown in the literature that the use of interactive quantitative CAD with scoring does not result in any significant increase in radiologist reading time [19].

Our economic analysis is based on an assumption of 100,000 mammograms per year, which our practice exceeds. Extrapolated data shows 589 h per year of radiologist reading time spent on reviewing false marks from CAD whereas significantly less time (215 h) would result from false marks from AI-CAD. That equates to a potential time savings of 64% or 375 h of radiologist reading time per year. Using the data referenced above, we calculate that up to 10% more screening mammograms could be read with the saved time using AI-CAD instead of CAD which would result in 41,469 additional relative value units (RVUs) per year. This calculation is based on a 2017 RVU for a 2D screening mammogram of 3.85. Using the 2017 Medicare fee schedule of a global reimbursement of $138 per screening mammogram with CAD, as much as $1,488,362 per year of increased global revenue may be realized.

Limitations of this study are its retrospective design and relatively small sample size and small number of cancer cases (this was not a cancer enriched data set). Therefore, an ROC analysis of CAD and AI-CAD was not performed. Because of the small sample size of the data set, the sensitivity results of both CAD and AI-CAD is felt to be overestimated compared to a clinical setting. Future studies are planned to further evaluate sensitivity and specificity metrics.

**Fig. 6:**
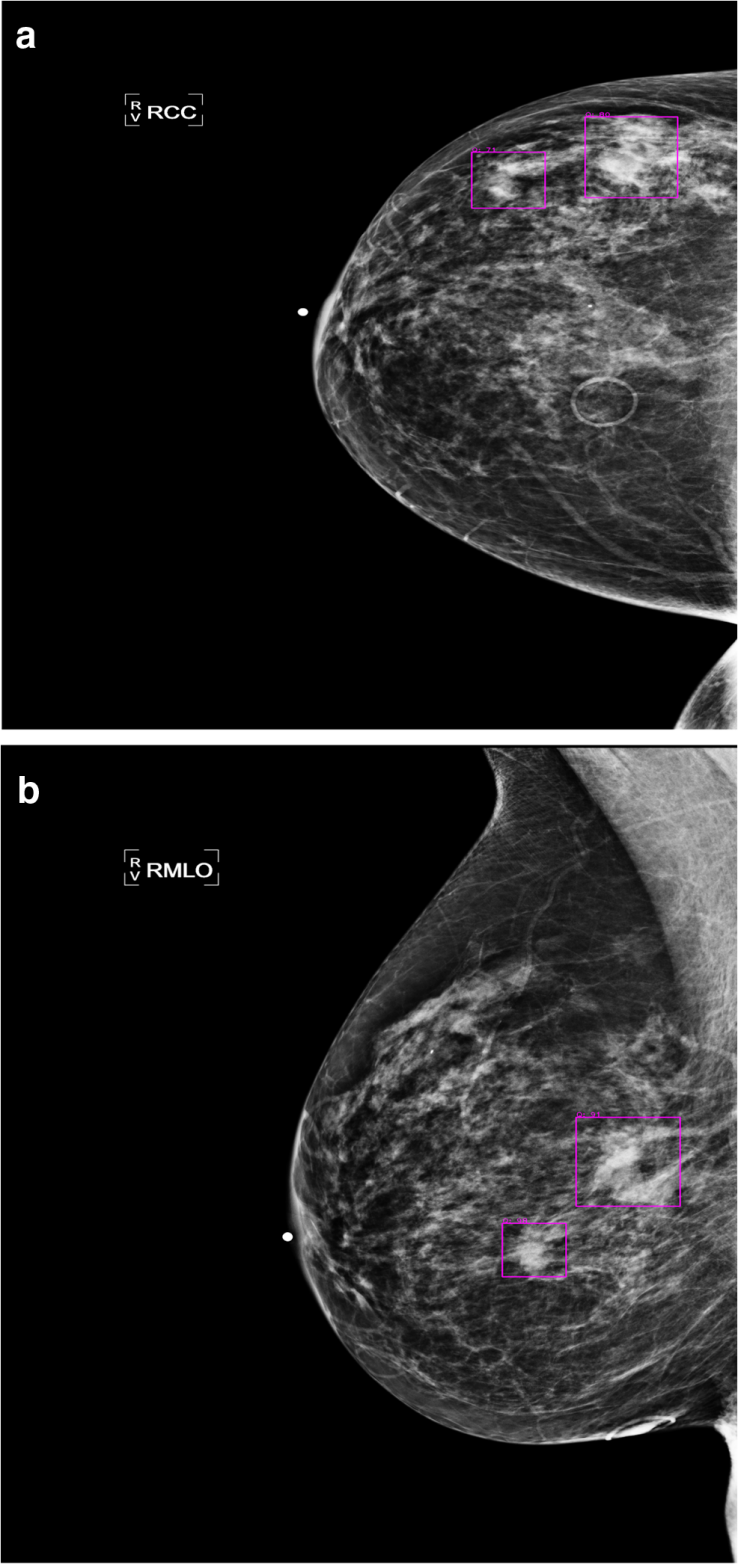
**a**, **b** Right CC and MLO screening mammogram AI-CAD mark-up images. These true positive markings of AI-CAD (pink squares) are each given a neuScore ™, which is a quantitative score for probability of malignancy, which ranges from 0 to 100. In this case, the scores of different marks ranged from 71 to 98. Invasive ductal cancer was confirmed at both sites

Aside from objective performance metrics, an important operational point of distinction between CAD and AI-CAD is that CAD works on raw DICOM data only. Although raw DICOM contains more information than for-presentation (processed) DICOM, it does have several drawbacks. CAD is not vendor-agnostic; each CAD vendor has its own proprietary software for processing raw DICOM data. In addition, CAD cannot usually run on prior or outside studies since raw DICOM data is typically not archived due to expense of the digital storage space. CAD requires pre-calibration with the image acquisition equipment. In contrast, AI-CAD is vendor agnostic, runs on processed images without requiring the raw DICOM data, and does not require calibration with the mammography equipment. These features make AI-CAD far more user-friendly and cost-effective, and allows for AI-CAD to be run on all prior and outside mammograms since raw data is not transferred and is not saved indefinitely. The quantitation of individually marked lesions through the use of deep learning techniques gives AI-CAD the additional benefit of providing probability of malignancy (neuScore ™) which CAD cannot do. An example of high neuScore ™ in a biopsy-proven cancer case is demonstrated in Fig. 6.

## Abbreviations

AI: Artificial intelligence
AI-CAD: Artificial intelligence-based computer-aided detection
BI-RADS: Breast Imaging Reporting and Data System
CAD: Computer-aided detection
CI: Confidence interval
DICOM: Digital Imaging and Communications in Medicine
FFDM: Full-field digital mammograms
FPPI: False-positives per image
FPR: False-positive rate
MQSA: Mammography Quality Standards Act
